# A Single NPFR Neuropeptide F Receptor Neuron That Regulates Thirst Behaviors in *Drosophila*

**DOI:** 10.1523/ENEURO.0259-25.2025

**Published:** 2025-10-23

**Authors:** Donnoban Orozco Ramirez, Brian P. Wang, Dan Landayan, Gagandeep Kaur, Fred W. Wolf

**Affiliations:** ^1^Quantitative and Systems Biology Graduate Program, School of Natural Sciences, University of California, Merced, Merced, California 95343; ^2^Biological Sciences Undergraduate Program, School of Natural Sciences, University of California, Merced, Merced, California 95343; ^3^Department of Molecular and Cell Biology, School of Natural Sciences, University of California, Merced, Merced, California 95343

**Keywords:** behavior, circuits, *Drosophila*, genetics, neuropeptides, thirst

## Abstract

Thirst is a strongly motivated internal state that is represented in central brain circuits that are only partially understood. Water seeking is a discrete step of the thirst behavioral sequence that is amenable to uncovering the mechanisms for motivational properties such as goal-oriented behavior, value encoding, and behavioral competition. In *Drosophila*, water seeking is regulated by the NPY-like neuropeptide NPF; however, the circuitry for NPF-dependent water seeking is unknown. To uncover the downstream circuitry, we identified the NPF receptor NPFR and the neurons it is expressed in as being acutely critical for thirsty water seeking in males. Refinement of the NPFR pattern uncovered a role for a single neuron, the L1-l, in promoting thirsty water seeking. The L1-l neuron increases its activity in thirsty flies and is involved in the regulation of dopaminergic neurons in long-term memory formation. Thus, NPFR and its ligand NPF, already known for its role in feeding behavior, are also important for a second ingestive behavior.

## Graphical abstract

**Figure:**
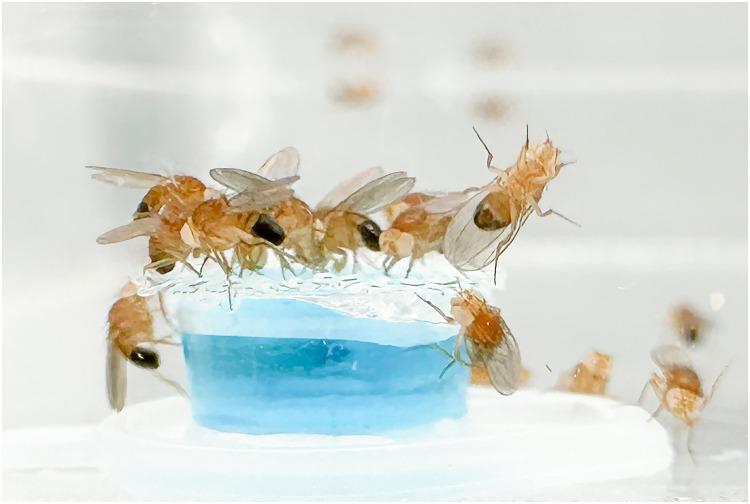
Visual Abstract

## Significance Statement

Understanding how a single motivated behavior is represented in the neural circuitry of the brain will help uncover drive-specific and universal encoding mechanisms for motivation. Thirst is useful because it is relatively simple compared with feeding behavior and it is strongly motivated. The fly *Drosophila*, with its straightforward genetics and complete connectome, is helpful in determining a complete circuit for thirsty water seeking. Here we discover a single neuron in the fly brain, the L1-l, that actively receives input from the NPY-like neuropeptide NPF to promote water seeking. Prior findings suggest that the L1-l modulates valence inputs into sensory processing centers, suggesting a similar function in thirsty seeking.

## Introduction

Thirst is an internal state that drives the seeking and consumption of water to restore osmotic homeostasis (Fitzsimons, 1972). The representation of thirst in the brain provides a means for understanding the neural encoding of motivational states, competition between conflicting needs, and behavioral sequencing. Uncovering the neural circuitry that supports specific steps of thirst behaviors will help reveal the bases for some of these complex computations. Animals become thirsty in a graded manner as their interstitial fluids increase in osmolarity. Thirst initiates a set of behaviors that often occur in sequence: searching for a source of water, determining if the water is palatable, water ingestion, and termination of water ingestion at repletion. It is accompanied by changes in sensory acuity, an escalating negative valence state and shorter duration positive valence anticipatory states, suppression of competing behaviors, and neuroendocrine signaling to minimize water loss (Betley et al., 2015; Gizowski et al., 2016; Gáliková et al., 2018; Gizowski and Bourque, 2018; Zandawala et al., 2021; Chu et al., 2024; Encarnacion-Rivera et al., 2025).

Thirsty *Drosophila* exhibit behaviors similar to other thirsty animals. Water deprivation switches humidity aversion to attraction, promotes directed flight and locomotion up humidity gradients, and increases water intake (Dethier and Evans, 1961; Ji and Zhu, 2015; Limbania et al., 2023). Osmosensory neurons detect osmotic changes in the circulating hemolymph and direct water ingestion through a simple circuit to neuroendocrine neurons in the pars intercerebralis (Jourjine, 2017; González Segarra et al., 2023). Humidity is detected by receptors on the antennae and humidity information is transmitted to a broad array of higher order central brain circuits (Enjin et al., 2016; Frank et al., 2017; Knecht et al., 2017; Marin et al., 2020; Chu et al., 2024). Thirst-dependent water seeking and humidity preference are controlled by central brain circuitry that includes neurons in the mushroom body learning and memory center, dopaminergic neurons that project to the mushroom body and provide valuation information, and a projection neuron that connects the primarily sensory subesophageal zone (SEZ) to the superior medial protocerebrum (SMP; Lin et al., 2014; Ji and Zhu, 2015; Landayan et al., 2021). The projection neuron, Janu-AstA, releases the Galanin/Kisspeptin/Spexin neuropeptide ortholog Allatostatin A (AstA) onto neurons that release the Neuropeptide Y (NPY) ortholog Neuropeptide F (NPF) to regulate thirsty water seeking.

These findings led us to search for the next downstream layer of the thirsty water seeking circuit by characterizing the role of NPFR, the only known receptor for NPF. NPFR regulates many processes in flies including food intake, energy homeostasis, the effect of temperature on circadian rhythms, courtship, stress, sensitivity to ethanol, and learning and memory (Wen et al., 2005; Wu et al., 2005; Krashes et al., 2009; Xu et al., 2010; Feng et al., 2021; Ryvkin et al., 2021, 2024; Yoshinari et al., 2021; Gao et al., 2024; Yuan et al., 2024). The interrelationship of these behaviors, if any, remains unclear. Assignment of specific functions of NPFR to specific neurons or cells can help untangle potential relationships and neural functions. For example, consolidation of long-term memory requires NPFR in the aversive-encoding PPL1 dopamine neurons, whereas circadian-dependent locomotor activity in the late afternoon is suppressed by NPFR in LPN neurons when it is cold, indicating separate functions of NPFR in these complex behaviors (Feng et al., 2021; Yuan et al., 2024). Here, we discovered that NPFR functions in a single neuron, the L1-l, to promote thirsty water seeking.

## Materials and Methods

### Genetics and culturing conditions

All strains were outcrossed for at least five generations to the *Berlin* strain of *Drosophila melanogaster* that contained the *w^1118^* mutation; this strain is indicated as “control” in the figures. Crosses were incubated at 25°C and 60% humidity on a 12 h light/dark schedule. Adult flies were removed from crosses after 2–3 d to create age-matched cohorts of progeny. Progeny were collected using CO_2_ anesthesia 2–4 d after eclosion and then held 1–2 d prior to testing. Male flies were used for all experiments. The experimenter was blinded to genotype for the experiment and its quantification. Strains and sources are listed in Table 1.

**Table 1.** Strains

Strain	Label	Source
*NPFR^sk8^*	*NPFR^sk8^*	Shu Kondo
*Dh44-Gal4*	*DH44-Gal4*	RRID:BDSC_51987
*NPF-T2A-DBD*	*NPF.DBD*	Ann-Shyn Chiang
*NPFR-Gal4*	*NPFR-Gal4*	Michael J. Texada
*R20A02-Gal4.AD*	*R20A02.AD*	RRID:BDSC_70582
*R23E12-Gal4.AD*	*R23E12.AD*	RRID:BDSC_70602
*R60E02-Gal4*	*R60E02*	RRID:BDSC_39250
*R60G05-Gal4*	*R60G05*	RRID:BDSC_39259
*R61H06-Gal4*	*R61H06*	RRID:BDSC_39281
*R61H06-Gal4.AD*	*R61H06.AD*	RRID:BDSC_70765
*R65C12-Gal4*	*R65C12*	RRID:BDSC_39348
*R65C12-Gal4.DBD*	*R65C12.DBD*	RRID:BDSC_68356
*UAS-Dcr-2*	*Dcr-2*	RRID:BDSC_24651
*UAS-MCFO-1*	*MCFO*	RRID:BDSC_64085
*10XUAS-IVS-myr::GFP*	*UAS-myr.GFP*	RRID:BDSC_32197
*UAS-NPFR RNAi*	*NPFR.IR*	RRID:VDRC_KK-112704
*UAS-Shibire^ts^*	*UAS-Shi^ts^*	RRID:BDSC_66599
*UAS-TeTx*	*UAS-TeTx*	Sean Sweeney
*UAS-TrpA1*	*UAS-TrpA1*	RRID:BDSC_26264

### Water seeking and foraging behaviors

Flies were deprived of water in culturing vials lined with a 2.5 × 15 cm strip of Whatman 3 MM filter paper that was infused with 5% sucrose and then dried. During the water deprivation period, the flies were held in an incubator at 25°C and 60% humidity (IN034, Darwin Chambers). Thin-walled Plexiglas behavioral chambers (IO Rodeo) were designed with two side-by-side arenas, each arena measuring 45 × 75 × 10 mm. The chamber and water source were acclimated to the testing temperature in a Peltier incubator (IN45, Torrey Pines Scientific). Groups of approximately 20 genetically identical flies (*n* = 1) were acclimated in the chambers for 10–15 min prior to introducing the water source. Humidity was monitored (EK-H4 multiplexer with SHT71 sensors, Sensirion) and maintained at 30–40% relative humidity. Flies were filmed from above with a Logitech C510 web camera at 30 fps with the arena placed on white light LED panel (Edmund Optics), such that flies appear as dark objects on a white background. Sources of water (containing 0.2% w/v erioglaucine, 229730050, Fisher Scientific) were presented to flies in an overturned cap of a 0.65 ml microcentrifuge tube. Water was made inaccessible by glueing (catalog #698, DAP) a 300 μm mesh grid (U-CMN-300-A, Component Supply) onto the cap. Flies were counted as occupying the water source if any part of their body overlapped with the water source. The number of flies occupying the water source was divided by the total number of flies in the arena to determine the fraction of flies on the source. Assessment of occupancy was made at 1, 2, 3, 4, 5, 7, 10, 13, 16, and 20 min by counting a single frame. Fractional occupancy was averaged for 7 and 10 min or for 10 and 13 min. Food foraging behavior followed the same protocol as for water seeking, except that flies were food deprived in culturing vials lined with Whatman 3MM soaked in water, and food-deprived flies were placed in the open field arena and presented with a small chunk of cane sugar (La Perruche Pure Cane Rough Cut Cubes) resting in an overturned 1.5 ml microcentrifuge tube cap. The two-choice experiment was the same as for water seeking, except that an inaccessible water source was placed right next to the dry sucrose source in the center of the arena. The preference index was calculated as the fraction of flies on the water source minus the fraction on the food source, averaged across the 7 and 10 min timepoints. Thermogenetic activation (*UAS-TrpA1*) and inhibition (*UAS-Shi^ts^*) of neurons was achieved by maintaining the flies at 18°C prior to the experiment to keep the thermogenetic tools off, introducing the flies into the testing chamber previously acclimated to the activating (29°C, TrpA1) and inhibiting (31°C, Shi^ts^) temperature for 15 min to acclimate the flies.

### Water intake

Flies were glued to microscope slides with clear nail polish while cold anesthetized and allowed to recover at 25°C and 60% humidity for 1–2 h. Consumption time was measured by repeatedly presenting the proboscis with a water drop extruded from the end of a 5 μl calibrated micropipette (53432-706, VWR), until flies failed to extend the proboscis to four sequential presentations. 0.2% w/v erioglaucine was added to help with quantification. Ingestion behavior was recorded on a stereoscope with a AmScope MD500 microscope eyepiece camera at 30 fps at 640 × 480. Pumping rate was determined with manual counts of video recordings of pharyngeal contractions during the initial 10 s of the first engagement, or shorter if the flies disengaged earlier.

### Immunohistochemistry

Adult brains were dissected in 1× phosphate-buffered saline (PBS; BP399, Fisher Scientific) containing 0.05% Triton X-100 (X100-500ML, Sigma-Aldrich; 0.05% PBT), fixed overnight at 4°C in 0.05% PBT with 2% paraformaldehyde (50-980-493, Fischer Scientific). Brains were then washed with 0.1% PBT 5 times for 20 min at room temperature, blocked in 0.5% PBT with 5% normal goat serum (733250, Lampire Biological Laboratories) and 0.5% bovine serum albumin (A7906-50G, Sigma-Aldrich) for 2 h at room temperature, and incubated with primary antibody diluted in block for 1–3 d at 4°C. Brains were washed with 0.1% PBT for five times for 20 min at room temperature, incubated in secondary antibody diluted in 0.1% PBT for 1–2 d at 4°C, washed with 0.1% PBT for five times for 20 min at room temperature, and equilibrated into Vectashield (H-1000, Vector Laboratories) overnight at 4°C. Samples were mounted on slides and imaged on a Zeiss LSM-880 confocal microscope with a 20× objective. Image stacks were processed in Fiji, and brightness and contrast were adjusted in Adobe Photoshop. For MultiColor FlpOut experiments, flies were heat shocked in a 37°C water bath for 40–45 min before resting at 25°C and 60% humidity for 2 d, and then dissected and stained as above. Antibodies used were rabbit anti-GFP, 1:1,000 (A6455, Invitrogen/Fisher); chicken anti-GFP, 1:1,000 (AB13970, Abcam); mouse anti-Bruchpilot, 1:25 (NC82, Developmental Studies Hybridoma Bank); rabbit anti-NPFR, N-terminal, 1:25 (RB-19-0003-100, RayBiotech); goat anti-rabbit Alexa Fluor 488, 1:350 (A11034, Molecular Probes); goat anti-mouse Alexa Fluor 594, 1:350 (A11032, Molecular Probes); donkey anti-chicken FITC, 1:350 (SA172000, Pierce); and goat anti-rabbit Alexa Fluor 594, 1:350 (A11037, Molecular Probes).

### Experimental design and statistical analysis

Groups of 20 genetically identical flies of the same age constituted an *n* = 1 for all behavioral assays, except for water intake where individual animals were assessed. No statistical methods were used to predetermine sample sizes. An *n* = 8 was previously determined to give discriminative power for the water seeking and food foraging open field assays (Landayan et al., 2021). For water intake an *n* = 20–30 individuals follows the design in prior reports (Jourjine, 2017). Experiments were typically performed with flies from two to three separate crosses and across 2–3 d. Statistical analysis was carried out in Prism v10.5.0. All statistical tests and outcomes are detailed in Table 2. Dots overlaid on bar graphs are the value measured for individual groups, or for individuals for water intake. No data was excluded using statistical methods. Error bars are the standard error of the mean, except for water intake, where data is represented as box and whiskers that represent the minimum and maximum values.

**Table 2.** Genotypes and statistical analyses

Figure	Measurement	#	Genotype/condition	*n*	Statistics test	Post hoc test	Result	Adjusted *p*
1*B*	Thirsty seeking, inaccessible	1	*NPFR^sk8^*	8	Unpaired *t* test (two-tailed)	*F* test, *p* = 0.7384	**	0.0044 (1 vs 2)
		2	*w^1118^*	8				
	Thirsty seeking, accessible	3	*NPFR^sk8^*	8	Unpaired *t* test (two-tailed)	*F* test, *p* = 0.4732	ns	0.1612 (3 vs 4)
		4	*w^1118^*	8				
1*C*	Thirsty water intake, total time	1	*NPFR^sk8^*	23	Mann–Whitney test (two-tailed)		****	<0.0001 (1 vs 2)
		2	*w^1118^*	28				
	Thirsty water intake, pump frequency	3	*NPFR^sk8^*	23	Unpaired *t* test (two-tailed)	*F* test, *p* = 0.4025	****	<0.0001 (3 vs 4)
		4	*w^1118^*	28				
1*E*	Thirsty seeking, inaccessible	1	*NPFR-Gal4 > UAS-NPFR.IR,UAS-Dcr-2*	8	One-way ANOVA	Holm–Šídák's		
		2	*NPFR-Gal4>+*	8			***	0.0006 (1 vs 2)
		3	*+>UAS-NPFR.IR,UAS-Dcr-2*	7			****	<0.0001 (1 vs 3)
2*A*	Thirsty seeking, inaccessible	1	*NPFR-Gal4 > UAS-TeTx*	8	Kruskal–Wallis	Dunn's		
		2	*NPFR-Gal4>+*	8			***	0.0001 (1 vs 2)
		3	*+>UAS-TeTx*	8			*	0.0427 (1 vs 3)
2*B*	Thirsty seeking, inaccessible	1	*NPFR-Gal4 > UAS-Shi^ts^*	8	One-way ANOVA	Holm–Šídák's		
		2	*NPFR-Gal4>+*	8			***	0.0007 (1 vs 2)
		3	*+>UAS-Shi^ts^*	8			**	0.0016 (1 vs 3)
2*C*	Thirsty seeking, inaccessible	1	*NPFR-Gal4 > UAS-TrpA1*	16	Brown–Forsythe	Dunnett's T3		
		2	*NPFR-Gal4>+*	12			****	<0.0001 (1 vs 2)
		3	*+>UAS-TrpA1*	12			****	<0.0001 (1 vs 3)
	Thirsty seeking, accessible	4	*NPFR-Gal4 > UAS-TrpA1*	9	One-way ANOVA	Holm–Šídák's		
		5	*NPFR-Gal4>+*	8			****	<0.0001 (4 vs 5)
		6	*+>UAS-TrpA1*	8			****	<0.0001 (4 vs 6)
2*D*	Hungry seeking	1	*NPFR^sk8^*	8	Mann–Whitney test (two-tailed)		***	0.0002 (1 vs 2)
		2	*w^1118^*	8				
2*E*	Hungry seeking	1	*NPFR-Gal4 > UAS-TrpA1*	8	One-way ANOVA	Holm–Šídák's		
		2	*NPFR-Gal4>+*	8			***	0.0003 (1 vs 2)
		3	*+>UAS-TrpA1*	8			****	<0.0001 (1 vs 3)
2*F*	Hungry two-choice	1	*NPFR-Gal4 > UAS-TrpA1*	8	Wilcoxon signed rank test		**	0.0078
		2	*NPFR-Gal4>+*	8			**	0.0039
		3	*+>UAS-TrpA1*	8			**	0.0020
3*B*	Thirsty seeking, inaccessible	1	*R61H06-Gal4 > UAS-NPFR.IR,UAS-Dcr-2*	8	One-way ANOVA	Holm–Šídák's		
		2	*R61H06-Gal4>+*	8			****	<0.0001 (1 vs 2)
		3	*+>UAS-NPFR.IR,UAS-Dcr-2*	8			**	0.0016 (1 vs 3)
		4	*R60G05-Gal4 > UAS-NPFR.IR,UAS-Dcr-2*	7	One-way ANOVA	Holm–Šídák's		
		5	*R60G05-Gal4>+*	8			****	<0.0001 (4 vs 5)
		6	*+>UAS-NPFR.IR,UAS-Dcr-2*	8			**	0.0046 (4 vs 6)
		7	*R65C12-Gal4 > UAS-NPFR.IR,UAS-Dcr-2*	8	Kruskal–Wallis	Dunn's		
		8	*R65C12-Gal4>+*	9			**	0.0053 (7 vs 8)
		9	*+>UAS-NPFR.IR,UAS-Dcr-2*	9			*	0.0143 (7 vs 9)
		10	*R60E02-Gal4 > UAS-NPFR.IR,UAS-Dcr-2*	8	One-way ANOVA	*p* = 0.1677		
		11	*R60E02-Gal4>+*	8				
		12	*+>UAS-NPFR.IR,UAS-Dcr-2*	8				
3*C*	Thirsty seeking, inaccessible	1	*R61H06-Gal4.AD;R65C12-Gal4.DBD > UAS-NPFR.IR,UAS-Dcr-2*	8	One-way ANOVA	Holm–Šídák's		
		2	*R61H06-Gal4.AD;R65C12-Gal4.DBD>+*	8			**	0.0077 (1 vs 2)
		3	*+>UAS-NPFR.IR,UAS-Dcr-2*	8			****	<0.0001 (1 vs 3)
		4	*R61H06-Gal4.AD;R65C12-Gal4.DBD > UAS-TeTx*	6	One-way ANOVA	Holm–Šídák's		
		5	*R61H06-Gal4.AD;R65C12-Gal4.DBD>+*	8			*	0.0180 (4 vs 5)
		6	*+>UAS-TeTx*	5			***	0.0002 (4 vs 6)
5*A’*	Thirsty seeking, accessible	1	*R20A02-Gal4.AD;NPF-Gal4.DBD > UAS-NPFR.IR,UAS-Dcr-2*	11	One-way ANOVA	Holm–Šídák's		
		2	*R20A02-Gal4.AD;NPF-Gal4.DBD>+*	10			*	0.0177 (1 vs 2)
		3	*+>UAS-NPFR.IR,UAS-Dcr-2*	10			*	0.0269 (1 vs 3)
5*B’*	Thirsty seeking, accessible	1	*R23E12-Gal4.AD;NPF-Gal4.DBD > UAS-NPFR.IR,UAS-Dcr-2*	8	One-way ANOVA	Holm–Šídák's		
		2	*R23E12-Gal4.AD;NPF-Gal4.DBD>+*	8			**	0.0015 (1 vs 2)
		3	*+>UAS-NPFR.IR,UAS-Dcr-2*	8			ns	0.2554 (1 vs 3)
5*C’*	Thirsty seeking, accessible	1	*Dh44 > UAS-NPFR.IR,UAS-Dcr-2*	8	One-way ANOVA	*p* = 0.8742		
		2	*Dh44-Gal4>+*	8				
		3	*+>UAS-NPFR.IR,UAS-Dcr-2*	8				

*p < 0.05, **p < 0.01, ***p < 0.001, ****p < 0.0001.

## Results

### NPFR acts in NPFR-Gal4 neurons to promote thirsty water seeking and intake

**Figure 1.**
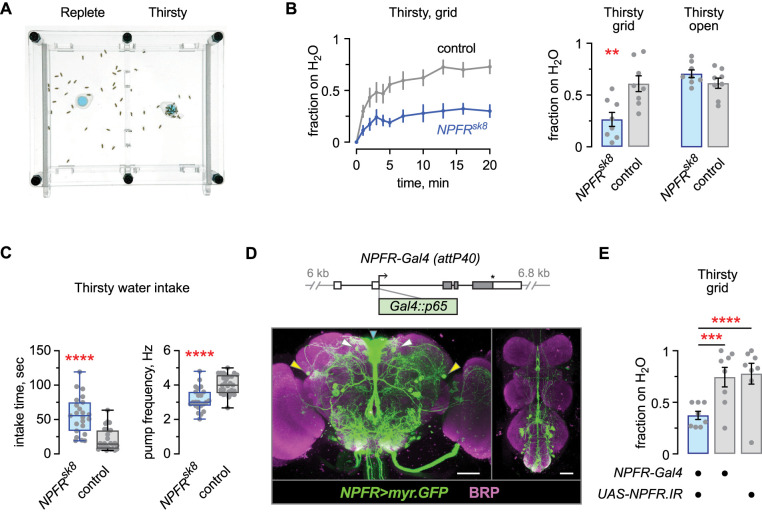
NPFR regulates thirst behaviors in *NPFR-Gal4* neurons. ***A***, Open field device for assessing water seeking, with an inaccessible water source placed at the center of each arena. ***B***, Left, Time course of flies occupying a gridded inaccessible water source over time, genetic control flies, and *NPFR^sk8^* strong loss-of-function mutants. Flies were water deprived on dry sucrose for 16 h at 25°C and 60% humidity. Right, *NPFR^sk8^* mutants exhibit decreased thirsty water seeking specifically to an inaccessible source. Left, Unpaired *t* test, two-tailed, *p* = 0.0044; right: Unpaired *t* test, two-tailed, *p* = 0.1612. ***C***, Increased intake time and decreased pharyngeal pumping rate in *NPFR^sk8^* mutants. Left, Mann–Whitney test, two-tailed, *p* < 0.0001; right, Unpaired *t* test, two-tailed, *p* < 0.0001. ***D***, Genomic structure (top) and expression (bottom) of *NPFR-Gal4* in the *Drosophila* central brain (left) and ventral nerve cord (right), immunostained for GFP and the presynaptic protein Bruchpilot (BRP) to reveal the synaptic neuropil. Arrowheads point to the L1-l (yellow), P1 (white), and pars intercerebralis (cyan) neurons. Scale bars, 50 μm. ***E***, *NPFR RNAi* (*UAS-NPFR.IR,UAS-Dcr-2*) in *NPFR-Gal4* reduced thirsty water seeking. One-way ANOVA with Holm–Šídák's multiple comparisons test; *Gal4/UAS* versus *Gal4*, *p* = 0.0006; *Gal4/UAS* versus *UAS*, *p* < 0.0001.

To ask if NPFR functions in thirst, we tested a loss-of-function *NPFR* mutant for thirsty water seeking. *NPFR^sk8^* is a frameshift mutation near the 5′ end of the *NPFR* open reading frame that eliminates NPFR expression (Ameku et al., 2018). We measured water seeking in an open field assay, where a group of 20 flies are placed in an arena with a small reservoir of water that is made inaccessible by placing a mesh grid over the reservoir (Fig. 1*A*). The reservoir creates a shallow humidity gradient, and thirsty flies travel up the gradient to occupy the inaccessible water source in a water deprivation- and time-dependent manner. *NPFR* mutant flies showed reduced water seeking when thirsty (Fig. 1*B*). In contrast, *NPFR* mutants performed normally when given an open, accessible water source, indicating that *NPFR* mutants develop the internal state of thirst (Fig. 1*B*). To determine if NPFR regulates thirst broadly or is specific to the water seeking step, we tested water ingestion by presenting flies with a small drop of water on their proboscis, measuring ingestion time and pharyngeal pumping frequency. Thirsty *NPFR* mutants drank for a longer time and had a decreased pumping frequency (Fig. 1*C*). Thus, flies lacking NPFR have blunted water seeking, and apparent compensatory changes in water intake time and rate. To identify the site of NPFR action, we asked if *NPFR* acts in neurons labeled by an *NPFR-Gal4* transgene. In this transgene *Gal4* is inserted immediately 3′ to the NPFR initiator methionine in an 18 kb genomic clone of the *NPFR* locus that is integrated into the genome at the *attP40* landing site (Fig. 1*D*). *NPFR-Gal4* driving expression of myristoylated GFP (*UAS-myr.GFP*) revealed expression in ∼100 neurons. The pattern included the NPF-positive neurons L1-l and P1, neuroendocrine cells in the pars intercerebralis, additional unidentified central brain neurons, and ∼20 neurons in the ventral nerve cord (Fig. 1*D*). When *NPFR* RNAi (*UAS-NPFR.IR*) was driven by *NPFR-Gal4*, thirsty flies showed reduced water seeking (Fig. 1*E*). Thus, NPFR acts in neurons in the *NPFR-Gal4* pattern to promote thirsty water seeking.

### Activity of NPFR-Gal4 neurons is acutely required for thirsty water seeking and for food foraging

**Figure 2.**
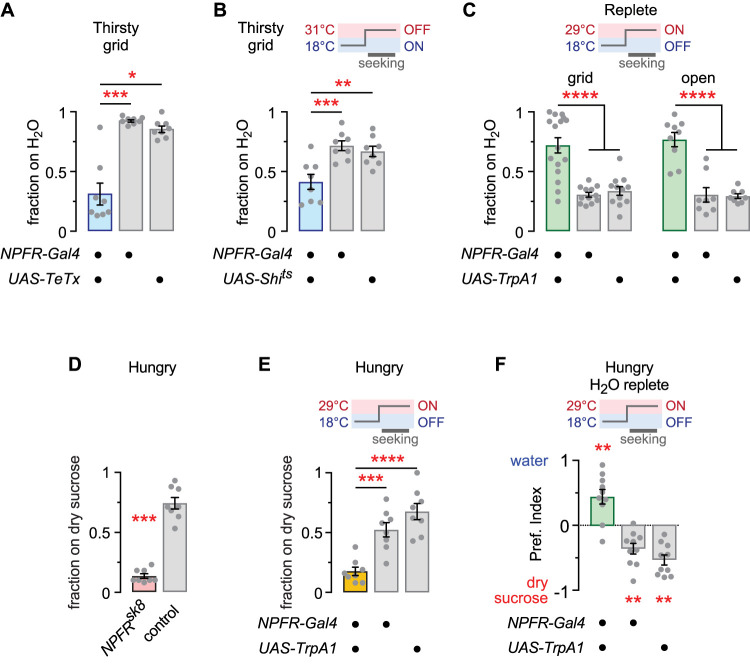
NPFR neuron activity is sufficient to promote water seeking and to regulate feeding behavior. ***A***, NPFR neuron inactivation with the tetanus toxin light chain (*UAS-TeTx*) blocks thirsty water seeking. Kruskal–Wallis ANOVA with Dunn’s multiple comparisons test; *Gal4/UAS* versus *Gal4*, *p* = 0.0001; *Gal4/UAS* versus *UAS*, *p* = 0.0427. ***B***, Acute synaptic silencing (*UAS-Shi^ts^*) of NPFR neurons decreases thirsty water seeking. One-way ANOVA with Holm–Šídák's multiple comparisons test; *Gal4/UAS* versus *Gal4*, *p* = 0.0007; *Gal4/UAS* versus *UAS*, *p* = 0.0016. ***C***, Acute depolarization (*UAS-TrpA1*) of NPFR neurons increases water seeking in water-replete flies, with a gridded or an open water source. Left, Brown–Forsythe ANOVA with Dunnett’s T3 multiple comparisons test; *Gal4/UAS* versus *Gal4*, *p* < 0.0001; *Gal4/UAS* versus *UAS*, *p* < 0.0001. Right, One-way ANOVA with Holm–Šídák's multiple comparisons test; *Gal4/UAS* versus *Gal4*, *p* < 0.0001; *Gal4/UAS* versus *UAS*, *p* < 0.0001. ***D***, *NPFR^sk8^* mutant flies fail to forage for food when wet starved. Mann–Whitney test, two-tailed, *p* = 0.0002. ***E***, Acute depolarization (*UAS-TrpA1*) of NPFR neurons in wet starved flies decreases foraging for food. One-way ANOVA with Holm–Šídák's multiple comparisons test; *Gal4/UAS* versus *Gal4*, *p* = 0.0003; *Gal4/UAS* versus *UAS*, *p* < 0.0001. ***F***, Hungry but water-replete (wet starved) flies given a choice between dry sucrose and water choose water when NPFR neurons are activated. Wilcoxon signed rank test. *Gal4*/*UAS*, *p* = 0.0078; *Gal4*, *p* = 0.0039; *UAS*, *p* = 0.0020.

We next determined the role of neuronal activity in the *NPFR-Gal4* pattern. We inactivated the neurons in two ways. First, expression of the tetanus toxin light chain (*UAS-TeTx*) that blocks presynaptic release of synaptic vesicles in *NPFR-Gal4* reduced thirsty water seeking (Fig. 2*A*). Second, acute and rapid inactivation of NPFR neurons using a temperature sensitive dominant negative Shibire (dynamin, *UAS-Shi^ts^*) also reduced thirsty water seeking (Fig. 2*B*). Thus, NPFR neurons promote thirsty water seeking during the water seeking task. Finally, to test if NPFR neurons are sufficient to promote water seeking, we acutely and rapidly activated them using the TrpA1 cation channel (*UAS-TrpA1*) in water-replete flies. NPFR neuron activation increased water seeking behavior (Fig. 2*C*). Thus, NPFR neurons are both necessary and sufficient for water seeking behavior, and they regulate water seeking via the NPFR receptor. NPF regulates both feeding and thirst-driven behavior (Beshel and Zhong, 2013; Landayan et al., 2021). We found that hungry *NPFR* mutants had a strong deficit in foraging for dry sucrose (Fig. 2*D*). Acute TrpA1 activation of NPFR neurons also strongly reduced feeding behavior (Fig. 2*E*). Since activation of NPFR neurons promoted water seeking but inhibited feeding behavior, we predicted that hungry flies would prefer water seeking when NPFR neurons are activated. Hungry but water-replete *NPFR* > *TrpA1* flies given a choice between water and dry sucrose preferred the water source (Fig. 2*F*). Thus, ongoing activity of the NPFR neurons promotes water seeking and inhibits feeding behavior, even when water seeking is mismatched to the prevailing internal state.

### A subset of NPFR neurons promotes thirsty water seeking

**Figure 3.**
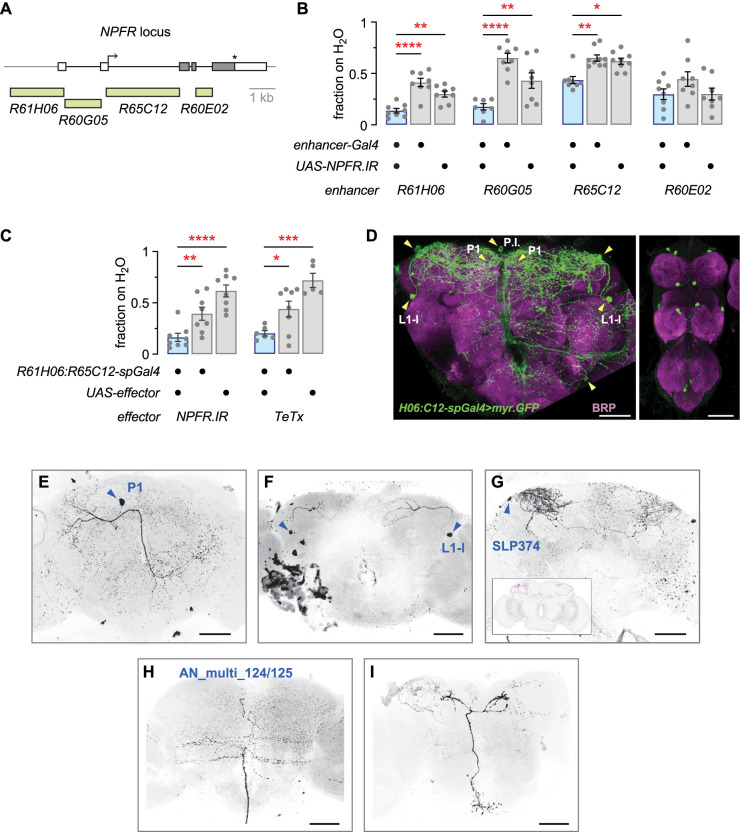
A subset of NPFR neurons promote thirsty water seeking. ***A***, Genomic location of the DNA fragments used to create NPFR *enhancer-Gal4s*. ***B***, Effect of reducing NPFR expression in the NPFR *enhancer-Gal4s*. Left, One-way ANOVA with Holm–Šídák's multiple comparisons test; *Gal4/UAS* versus *Gal4*, *p* < 0.0001; *Gal4/UAS* versus *UAS*, *p* = 0.0016. Center left, One-way ANOVA with Holm–Šídák's multiple comparisons test; *Gal4/UAS* versus *Gal4*, *p* < 0.0001; *Gal4/UAS* versus *UAS*, *p* = 0.0046. Center right, Kruskal–Wallis with Dunn’s multiple comparisons test; *Gal4/UAS* versus *Gal4*, *p* = 0.0053; *Gal4/UAS* versus *UAS*, *p* = 0.0143. Right, One-way ANOVA, *p* = 0.1677. ***C***, Effect of *NPFR RNAi* (left) and *TeTx* (right) on thirsty water seeking when expressed in the *R61H06-Gal4.AD*;*R65C12-Gal4.DBD split-Gal4*. Left, One-way ANOVA with Holm–Šídák's multiple comparisons test; *spGal4/UAS* versus *spGal4*, *p* = 0.0077; *spGal4/UAS* versus *UAS*, *p* < 0.0001. Right, One-way ANOVA with Holm–Šídák's multiple comparisons test; *spGal4/UAS* versus *spGal4*, *p* = 0.0180; *spGal4/UAS* versus *UAS*, *p* = 0.0002. ***D***, Expression pattern of *R61H06*:*R65C12*-*spGal4* in the central brain (left) and ventral nerve cord (right). Arrowheads point to cell bodies. Scale bars, 50 μm. ***E–I***, Multicolor flip-out (MCFO) stochastic labeling reveals morphology of individual neurons in *R61H06*:*R65C12*-*spGal4*. Inset in ***G*** is the morphology of SLP374 in the FAFB. Arrowheads point to cell bodies. Scale bars, 50 μm.

To begin to identify the specific NPFR neurons that control thirsty seeking, we reduced NPFR expression in neurons that are labeled in four different *NPFR* genomic *enhancer-Gal4s* (Fig. 3*A*). Three of the four, *R61H06-Gal4*, *R60G05-Gal4*, and *R65C12-Gal4*, resulted in reduced thirsty water seeking when driving *NPFR* RNAi (Fig. 3*B*). Previously available whole-brain immunohistochemical analysis revealed that each of these *enhancer-Gal4s* labeled complex patterns of neurons in the central brain (Jenett et al., 2012). We were unable to visually identify neurons that were common between all three *enhancer-Gal4s*. To narrow the potential site of action for NPFR, we employed *split-Gal4* technology, where the activation domain of *Gal4* was driven by the *R61H06* enhancer fragment, and the DNA-binding domain of *Gal4* was driven by the *R65C12* enhancer fragment. Reagents to generate *split-Gal4s* with *R60G05* were not available. Expression of either *NPFR* RNAi or TeTx with *R61H06:R65C12-spGal4* resulted in reduced thirsty water seeking (Fig. 3*C*). Thus, NPFR water seeking neurons exist in the *R61H06:R65C12-spGal4* pattern. This *spGal4* was expressed in 5–7 pairs of neurons in the central brain and 10–15 pairs of neurons in the ventral nerve cord (Fig. 3*D*). To better identify the neurons in the central brain, we used the multicolor flip out (MCFO) technique to stochastically label neuron subsets (Fig. 3*E–I*). This technique unambiguously identified the presence of the two NPF-positive NPFR neurons, the L1-l and the P1 neurons (Fig. 3*E*,*F*). The MCFO brains also revealed a dorsolateral neuron that is morphologically similar to SLP374 in the full adult female brain (FAFB) electron microscopy reconstruction and connectome (Dorkenwald et al., 2024; Schlegel et al., 2024; Fig. 3*G*). We also recovered ascending neurons that appear to be either AN_multi_124 or AN_multi_125 in the FAFB (Fig. 3*H*). Two neurons were present in the *spGal4* but were not recovered in the MCFO screen. First, 1–2 neurons in the pars intercerebralis were labeled (Fig. 3*D*). Second, a ventral lateral neuron was tentatively identified as DNg30, based on the position and large size of its cell body and its unique axonal projections.

### NPFR-positive neurons in the NPFR split-Gal4

**Figure 4.**
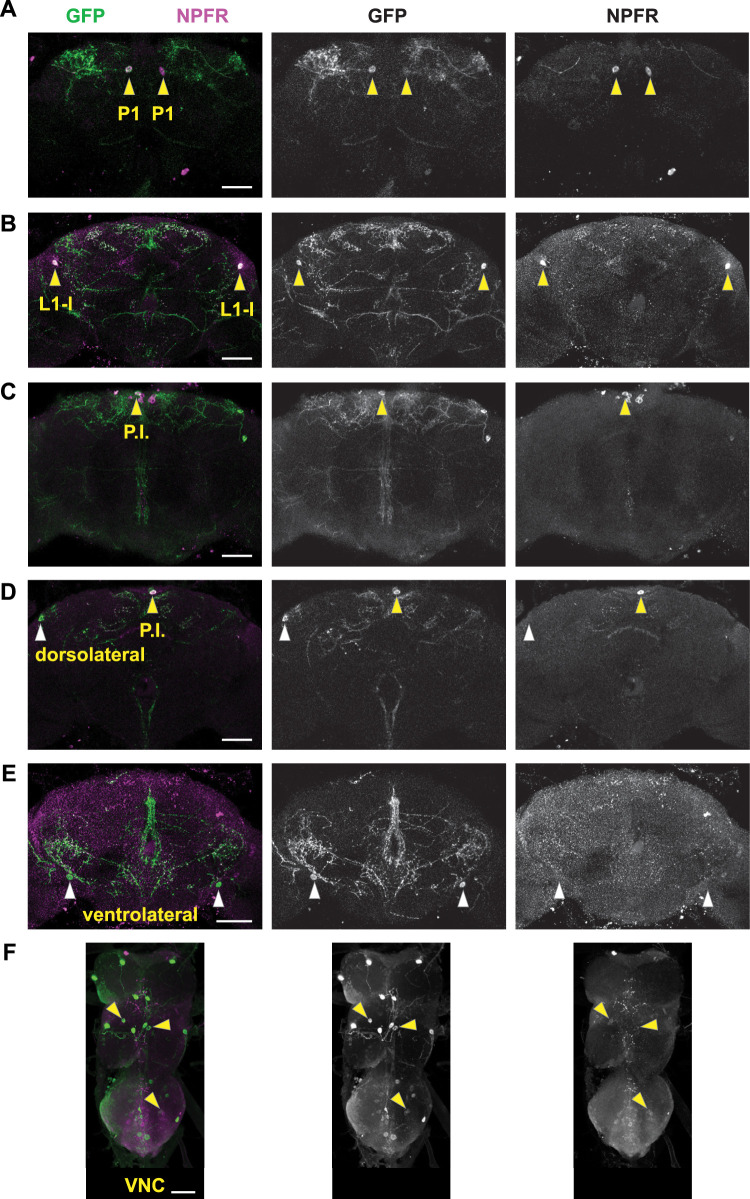
Immunostaining of *R61H06*:*R65C12*-*spGal4* > *UAS-myr.GFP* central brains (***A–E***) and ventral nerve cords (***F***) with antibodies to GFP (green) and NPFR (magenta). Each panel is a maximum intensity projection of a z-series to reveal individual cell bodies. Arrowheads point to positions of GFP-positive cell bodies that are positive (yellow) or negative (white) for NPFR. Scale bars, 50 μm.

We immunostained *R61H06:R65C12-spGal4* expressing myristoylated GFP with antibodies to GFP and NPFR to help reveal which neurons in the *spGal4* are NPFR-positive (Fig. 4). As expected, the L1-l and P1 neurons were NPFR-positive (Fig. 4*A*,*B*). Also NPFR-positive was the neuron in the pars intercerebralis (Fig. 4*C*,*D*). We were unable to detect NPFR in either of the other two central brain neurons, the putative SLP374 neuron and the putative DNg30 neuron (Fig. 4*D*,*E*). In the ventral nerve cord two pairs of as-yet unidentified neurons were weakly positive for NPFR (Fig. 4*F*).

### NPFR acts in the L1-l neuron to promote thirsty water seeking

**Figure 5.**
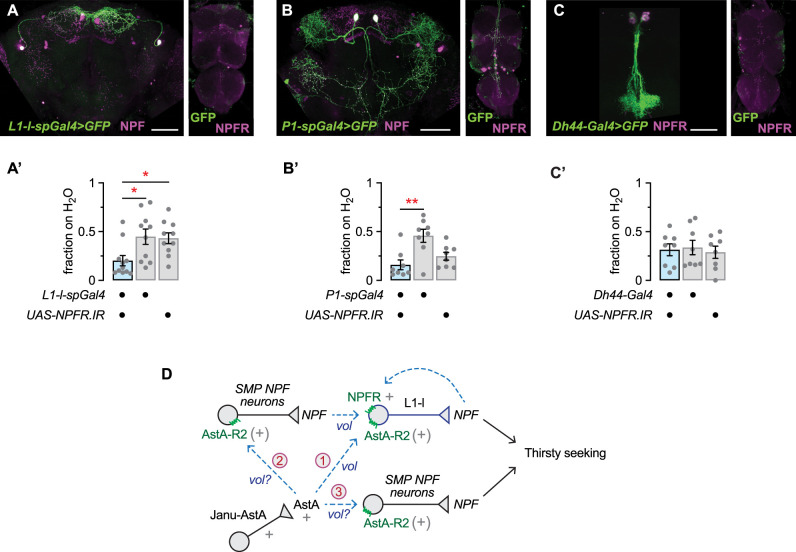
Effects on thirsty water seeking of reducing NPFR expression in individual NPFR neurons. ***A***, ***A’***, *L1-l-spGal4* (*R20A02-Gal4.AD*;*NPF-Gal4.DBD*) central brain neurons immunostained for GFP and NPF (***A***, left) and the ventral nerve cord immunostained for GFP and NPFR (***A***, right). Reduced thirsty water seeking with reduced NPFR expression in *L1-l-spGal4* neurons (***A’***). One-way ANOVA with Holm–Šídák's multiple comparisons test; *spGal4/UAS* versus *spGal4*, *p* = 0.0177; *spGal4/UAS* versus *UAS*, *p* = 0.0269. ***B***, ***B’***, *P1-spGal4* (*R23E12-Gal4.AD*;*NPF-Gal4.DBD*) neurons immunostained for GFP and NPF (***B***, left) and the ventral nerve cord immunostained for GFP and NPFR (***B***, right). Nonsignificant trend toward decreased thirsty water seeking with reduced NPFR expression in *P1-spGal4* neurons (***B’***). One-way ANOVA with Holm–Šídák's multiple comparisons test; *spGal4/UAS* versus *spGal4*, *p* = 0.0015; *spGal4/UAS* versus *UAS*, *p* = 0.2554. ***C***, ***C’***, *DH44-Gal4* neurons immunostained for GFP and NPFR (left, central brain; right, ventral nerve cord; ***C***). No effect on thirsty water seeking with reduced NPFR expression in *Dh44-Gal4* neurons (***C’***). One-way ANOVA, *p* = 0.8742. ***D***, Circuit models. “+” indicates characterized role, “(+)” indicates AstA-R2 functioning on NPF neurons, dashed arrows and “vol” indicate proposed volumetric transmission, and circled numbers designate the proposed models described in the Discussion. Scale bars, 50 μm.

Water deprivation increases neuronal activity in NPFR-positive L1-l and P1 neurons (Landayan et al., 2021). Neurons in the neuroendocrine pars intercerebralis regulate water ingestion, suggesting that they may also be a site of NPFR action for thirsty water seeking (González Segarra et al., 2023). We created new *spGal4s* to test the water seeking role of the L1-l and the P1 neurons. To do this, we used an *NPF-DBD* hemidriver and screened for *-AD* hemidrivers that together labeled small numbers or individual neurons. We identified *R20A02-AD;NPF-DBD* as an L1-l-specific driver and *R23E12-AD;NPF-DBD* as a P1-specific *spGal4* driver, with no additional labeled neurons in the ventral nerve cord (Fig. 5*A*,*B*). The P1 neurons are descending neurons with cell bodies in the central brain and a process that descends to the ventral nerve cord (Fig. 5*B*, right). Expression of NPFR RNAi in the L1-l neuron reduced thirsty water seeking (Fig. 5*A’*). Expression of NPFR RNAi in the P1 neuron caused a nonsignificant trend toward reduced water seeking in the P1 neuron (Fig. 5*B’*). Most or all NPFR-positive neurons in the pars intercerebralis are also positive for the neuropeptide DH44 that we confirmed (Fig. 5*C*; Ryvkin et al., 2024). Thus, we reduced NPFR expression in DH44 neurons with *Dh44-Gal4*; however, we did not observe an effect on thirsty water seeking (Fig. 5*C’*). Taken together, these results indicate that the L1-l neuron promotes water seeking by receiving NPF input through the NPFR receptor.

## Discussion

We identified NPFR, the receptor for the neuropeptide NPF, and neurons that express NPFR as critical for the motivated ingestive behaviors water seeking, water intake, and feeding. Using intersectional genetic techniques, we identified the bilaterally symmetric and NPF-positive L1-l neuron as requiring NPFR for the promotion of thirsty water seeking. Here we discuss the position of NPFR function in the emerging circuitry for innate thirst behavior in the *Drosophila* brain and how the L1-l neuron may integrate into brain circuits that regulate memories and the valuation of sensory input.

In mammals, the NPF ortholog NPY and its receptors are studied mainly for their role in suppressing hunger and modulating memory; however, there are a few reports that implicate NPY and the Y2 and Y4 NPY receptors in thirst (Morley and Flood, 1989; Wultsch et al., 2006). Hunger and thirst both strongly drive seeking and ingestion, and they interact behaviorally and at the neural circuit level in rodents (Augustine et al., 2020; Encarnacion-Rivera et al., 2025). Individual neurons in the *Drosophila* brain can regulate both hunger and thirst (Jourjine, 2017; Landayan et al., 2021; Shiu et al., 2022; González Segarra et al., 2023). NPF itself oppositely regulates hunger and thirst in flies (Landayan et al., 2021).

A Janu-AstA to NPF to NPFR circuit is supported by prior evidence: NPF functions downstream of Janu-AstA neurons via the AstA receptor AstA-R2 to regulate thirsty water seeking, and the L1-l neurons are activated by thirst (Landayan et al., 2021). Neither the available connectome reconstructions of the *Drosophila* brain nor anterograde synaptic tracing experiments support the L1-l as being directly postsynaptic to Janu-AstA (data not shown). Given these biological constraints, the following thirst circuits are possible (Fig. 5*D*). (1) AstA from Janu-AstA may function through volumetric transmission, also known as extrasynaptic diffusion, to reach NPFR expressing L1-l neurons within the SMP (Girven et al., 2022). Janu-AstA elaborates presynaptic release sites in the SMP, and L1-l neuronal processes pass through and near the Janu-AstA release zone. NPFR on the L1-l may receive autocrine NPF directly from the L1-l: AstA released from Janu-AstA neurons likely regulates the release of NPF from L1-l neurons. (2) An unidentified NPF neuron may function upstream of NPFR on the L1-l neurons, acting between Janu-AstA and L1-l. Three neurons synaptically connect Janu-AstA to the L1-l: MBON35 (receiving 24 or 3.8% of Janu-AstA synapses and delivering 75 or 4.2% of all synapses to the L1-l in the hemibrain), OviIN (receiving 28 or 4.5% of Janu-AstA synapses and delivering 34 or 1.9% synapses to the L1-l), and SMP081 (two per hemisphere, receiving 31–33 or 4.9–5.3% of Janu-AstA synapses and delivering 18–36 or 1–1.9% synapses to the L1-l). All three intermediate neuron types are considered “rich-club” neurons that make many synaptic connections across multiple brain regions. For example, MBON35 receives 21,335 synaptic inputs and delivers 1,746 synaptic outputs in the hemibrain. Moreover, none of these bridging neurons is likely to be NPF-positive, based on prior morphological characterization of the NPF neurons. Hence, in the second model volume transmission is likely necessary to allow for one of the other NPF neurons to bridge between Janu-AstA and L1-l. Candidate neurons based on their innervation of the SMP include the male-specific NPF-M neuron and the NPF-positive neurons in the evening groups of the circadian clock, the LNd2, LNd3, LNd6, and 5th LNv (Lee et al., 2006; Kim et al., 2013; Liu et al., 2019; Shafer et al., 2022). The small DMs are a final group of NPF neurons that arborize in the medial part of the dorsal protocerebrum; however, their identity in the connectome is not yet known. The small DMs encode positive valence (Shao et al., 2017). (3) Janu-AstA and L1-l may function in parallel circuits to promote thirsty water seeking. Finally, although NPFR neuron function is required and sufficient specifically during thirsty water seeking, and neuronal activity is increased in the L1-l by thirst, we did not explicitly test if NPFR itself functioned in development or adulthood.

What role might the L1-l play in thirsty seeking? The identity and function of L1-l output neurons can provide clues. In the FAFB there are only eight types of output neurons, with the left L1-l making a total of 37 synapses to postsynaptic neurons and the right 47. The two outputs receiving the most synapses from the L1-l are MBON35 (10 synapses per L1-l, FAFB) and SMP108 (9–12 synapses per L1-l, FAFB). Although a substantial number of L1-l output synapses are dedicated to the MBON35 and SMP108 (19–22 out of 50–55 total), the L1-ls represent only 0.12–0.15% of MBON35 or SMP108 inputs. Interestingly, all L1-l outputs are classified as “rich-club.” We suggest three possible interpretations. First, L1-ls signal a bias to “rich-club” neurons to generally tune or sensitize a brain state. For example, coincident activity of neurons from separate circuits onto a “rich-club” neuron may alter its likelihood of firing. This type of encoding could be part of internal state encoding where the influence of the state is graded over time to alter the probability of the execution of a thirst related behavior such as increased locomotion or sensory tuning. Second, the main targets of the L1-l may be due to volumetric transmission, rather than direct synaptic connections. Finally, it remains possible that existing connectomes do not capture important L1-l outputs.

The L1-l neuron is also named the DAL2 (dorsal anterior lateral) neuron, and it regulates long-term memory consolidation through its action on the PPL1 aversive dopamine neurons (Feng et al., 2021). Specifically, L1-l neurons inhibit the PPL1 neurons to decrease their interference with long-term memory consolidation. Despite no direct synaptic connectivity, L1-l release of NPF is critical for the repression of PPL1 neuron electrical activity through the NPFR receptor during memory consolidation. By analogy, elevated L1-l activity in the thirsty state may cause repression of PPL1 neurons, leading to changes in the sensitivity of the mushroom body circuitry to sensory inputs and positive valence dopaminergic inputs. Thirst permits appetitive long-term memory formation for water reward that is thought to involve specific sets of positive valence PAM dopaminergic neurons that innervate the mushroom body (Lin et al., 2024). A subset of positive valence PAM neurons are activated by water ingestion in thirsty flies and they regulate types of water-related memory formation (Lee et al., 2025). Similarly, foraging for food when hungry is regulated by NPFR on PPL1 neurons, suggesting that NPF-to-dopamine neuron signaling regulates a valence aspect of seeking related to ingestive behavior (Tsao et al., 2018). Finally, the L1-l output neuron SMP108 is presynaptic to the PAM reward dopamine neurons, and it functions in a type of learning termed second-order conditioning (Yamada et al., 2023).

## Synthesis

Reviewing Editor: Mihaela Iordanova, Concordia University - Loyola Campus Decisions are customarily a result of the Reviewing Editor and the peer reviewers coming together and discussing their recommendations until a consensus is reached. When revisions are invited, a fact-based synthesis statement explaining their decision and outlining what is needed to prepare a revision will be listed below. The following reviewer(s) agreed to reveal their identity: Suewei Lin.

The approaches used in this study are straightforward, and the results are generally convincing. The reviewers identified some concerns that are outlined below, which will strengthen the manuscript.

1. Please provide additional detail in the Methods section. For example, the water seeking and thermogenetic experiments are only described in general terms. The authors should clarify the specific timeline for temperature shifts and when the behavioral data were collected relative to this shift. Similarly, the two-choice assay presented in Figure 2F is not described in the Methods. A detailed explanation of how the assay was conducted, how preference was measured, and over what time period behavior was recorded is necessary. Finally, for the water seeking assay, more detail is needed on key parameters such as: how long behavior was recorded, when counting began, and what criteria were used to define a fly as being "on the water source."

2. Some clarification and supplementation of the data analyses are needed.

A) In Figure 1B (right), the authors conclude that NPFR mutant flies do not exhibit general behavioral deficits, based on their occupancy of an open, accessible water source at levels comparable to controls. However, this conclusion lacks sufficient support. First, the manuscript does not specify whether assay conditions, such as test duration and the timing of behavioral measurements, were consistent between the open and gridded water setups. This information is necessary for valid comparisons. More importantly, the open water assay does not effectively rule out locomotor impairments. Because flies can drink and remain at the source once they reach it, mutants with reduced mobility may still accumulate at the water source over time, resulting in occupancy levels similar to those of controls. To more directly assess general locomotor function, the authors should report metrics such as walking speed, and the duration and proportion of walking versus stopping bouts.

B) In Figure 5, the authors use split-Gal4 lines to isolate and manipulate specific brain neurons, including L1-l and P1, to support their claim that these neurons mediate thirsty water seeking. However, to convincingly argue that these split lines exclusively label brain neurons, the authors should include VNC staining data. This is particularly important given that previous figures show that the NPFR-Gal4 and related enhancer lines can drive expression in both brain and VNC neurons. Without VNC imaging for the split lines used in Figure 5, it is difficult to conclusively attribute behavioral effects solely to the targeted brain neurons.

C) The behavioral data from UAS-NPFR.IR control flies show notable variability, which complicates interpretation of the NPFR knockdown results. This is particularly evident in Figures 5A' and 5B', where the authors compare the effects of NPFR knockdown in L1-l and P1 neurons, respectively. While the authors report a significant reduction in water seeking for L1-l knockdown and only a non-significant trend for P1, it is important to note that the UAS-NPFR.IR control group in the L1-l experiment exhibited higher baseline water-seeking performance than the corresponding control in the P1 experiment (as well as that in the DH44 experiment in 5C'). This discrepancy raises the possibility that the observed "significant" reduction in L1-l may be partly driven by elevated performance in the control group, rather than a strong suppression in the experimental group. The authors should acknowledge this potential confound and consider whether the variability in control performance may affect the strength of their conclusions.

D) Following the previous point, at the end of the Results section, the authors state that "the effect on thirsty water seeking of NPFR knockdown in the L1-l was moderate compared to NPFR-Gal4," and use it to suggest that "additional NPFR neurons also support thirsty water seeking." This interpretation appears to be based on a comparison between Figure 1E (NPFR-Gal4) and Figure 5A' (L1-l). However, these experiments have different baselines, and no direct statistical comparison of effect sizes is provided. Given the variability in control group behavior and the absence of such a comparison, the conclusion is not currently well supported.

E) The NPFR knockdown experiments lack temporal control, leaving open the possibility that the observed behavioral effects stem from developmental alterations rather than an acute physiological role of NPFR in thirst regulation. The authors should acknowledge this caveat.

3. The visual abstract appears to be a photograph of the authors behavioral paradigm, and it does not seem to actually be a visual abstract. I would suggest moving this image into Fig. 1A.
